# Uncovering molecular abnormalities leading to the Warburg effect in primary refractory diffuse large B-cell lymphoma

**DOI:** 10.1038/bcj.2016.104

**Published:** 2016-12-02

**Authors:** M Soleja, M Mims, G Rivero

**Affiliations:** 1 Department of Internal Medicine, Baylor College of Medicine, Houston, TX, USA; 2 Section of Hematology/Oncology, Department of Internal Medicine and Dan L. Duncan Comprehensive Cancer Center at Baylor College of Medicine, Houston, TX, USA

For decades, it has been known that malignant cells have a propensity to metabolize glucose to lactate in the presence of oxygen.^1^ Normal lymphoid cells generate ATP from glycolysis and oxidative phosphorylation (OxPhos). After activation, B-lymphocytes proceed via differentiation programs to produce metabolically active subsets to ensure clonal expansion and antibody production. This activation process is highly dependent on B-cell-receptor (BCR) stimulation and upregulation of the glucose transporter Glut1.^2^ Glucose is metabolized to pyruvate, which is then fully oxidized by the Krebs cycle (Figure 1a). During malignant conversion, cells adopt metabolome resetting to optimize uncontrolled proliferation leading to increased glycolysis, hypoglycemia and augmented lactate production, a phenomenon normally described as Warburg effect (Figure 1b). Studies have described multiple pathways involved in the restructured metabolome; ^3, 4, 5^ however, there are limited clinical data tracing the phenomena of hypoglycemia with lactic acidosis back to these altered metabolic pathways. Here we present a patient with primary refractory diffuse large B-cell lymphoma (DLBCL) with severe hypoglycemia and lactic acidosis who succumbed to his disease. We highlight genomic alterations in our patient's tumor sample, which potentially cooperated to produce alterations in glucose metabolism and we describe evolving concepts that could facilitate treatment of this complication.

A 73-year-old White male with a history of chronic lymphocytic lymphoma (CLL) presented with an enlarging left neck mass and severe fatigue. The patient was originally diagnosed with CLL and treated with R-CHOP for six cycles in 2010. In 2012, he presented with multiple enlarged cervical lymph nodes. Biopsy showed high-grade histology consistent with transformation to DLBCL. He was enrolled in PI3K clinical trial, but did not respond.

Subsequently his PET CT showed intense uptake with maximum SUV of 23 (Figure 2a), in left submandibular and intrathoracic lymph nodes. A review of his 2012 biopsy showed large lymphoma cells positive for CD20, BCL2, MUM1 and PAX5, negative for CD10 and CD5, and variable BCL6 staining suggesting an activated B-cell (ABC) subtype. cMYC was positive in 60% of the malignant B cells. Bone marrow biopsy showed 25% involvement by lymphoma cells.

Metaphase karyotype revealed complex cytogenetics with 12 chromosomal abnormalities. Extracted DNA was tested with a custom-designed Leukemia Cancer Gene Mutation Panel using

AmpliSeq technology and showed *IDH2* c.419G>A (p.R140Q), *SRSF2* c.284C>G (p.P95R) and *TP* 53c.733 G>A (p.G245S) and c.380 C>T (p.S127F) mutations. The patient was initiated on salvage chemotherapy with Rituximab (R)–Bendamustine. Given immunohistochemistry suggestive of the ABC subtype associated with a high probability for activation of NfKB signaling, lenalidomide (LND) was added to his regimen.

On day 4 cycle 1 of R-Bendamustine, he presented to the hospital with loose stools, poor PO intake and pleural effusion. Empiric antibiotics including imipenem, vancomycin and micafungin were administered. Blood, urine, sputum and pleural fluid cultures were negative. Pleural fluid studies showed lymphoma cells. Lenalidomide was initiated on day five of hospitalization (day 9 of cycle 1 of BR) at 5 mg orally daily for 21 days on a 28-day cycle. On day 4 of lenalidomide (day 13 of cycle 1), a rapidly enlarging submental mass was detected on his PET CT (Figure 2a). Blood sugars were repeatedly below 60 mg/dl. Extensive infectious and endocrine evaluations were unrevealing. His oxygen saturation was 98%. His early morning cortisol was 18 μg/dl. ACTH stimulation test ruled out adrenal insufficiency. There was no evidence of liver failure or hypoproteinemia. His insulin, C-Peptide, IGF-2, sulfonyurea levels were all within appropriate range for degree of hypoglycemia. Aggressive glucose repletion with 20% dextrose solution at 100 cc/h intravenously and 50% dextrose boluses failed to resolve his hypoglycemia (Figure 2b). Refractory hypoglycemia persisted for days and serum lactic acid increased to as much as 13 mmol/l (Figure 2b). Given the patient's poor prognosis, his family decided to withdraw care and he expired from profound lactic acidosis. His refractory hypoglycemia and lactic acidosis in the absence of infection or endocrine abnormalities suggested the Warburg effect.

Lactic acid production in the absence of tissue oxygenation is categorized as type B lactic acidosis. However, Otto Warburg observed increased lactic acid production decades ago in cancer cells in normal oxygen concentrations. His interpretation of this observation was that cancer cells undergo a metabolic shift to produce ATP from glucose only via glycolysis without utilizing oxidative phosphorylation.^1^ Profound systemic lactic acidosis, as seen in our case, may be an extreme manifestation of the Warburg effect. Published cases have demonstrated extremely high mortality, but the few who survived achieved chemotherapy- induced tumor response.^6, 7, 8, 9^ There has been limited investigation as to why particular lymphomas demonstrate such a marked metabolic shift and whether genomic and oncogenic understanding could facilitate targeted interventions for this hematologic emergency.^6, 7, 8, 9^ In the case presented here, next-generation sequencing of unfractionated bone marrow DNA from our patient's lymphoma cells showed mutations in *IDH2*, and *TP53*. In addition, staining of his primary mass showed 60% MYC expression. IDH1/2 mutations have been primarily described in glioblastoma and myeloid neoplasms, but have also been reported in chondrosarcomas and angioimmunoblastic T-cell lymphoma.^3^ The *IDH2* mutation observed in this patient is associated with increased production of the oncometabolite 2-hydroxyglutarate (2-HG). Beside a well-established epigenetic role for 2-HG in tumorogenesis, the oncometabolite promotes glycolysis through inhibition of prolyl hydroxylase 2 (PHD2), an enzyme that normally functions to target hypoxia-inducible factor 1α (HIF-1α) to the proteosome.^3^ HIF-1 biases cellular metabolism toward glycolysis, but also enhances lactate production by inducing expression of lactate dehydrogenase-A.^3^ *In vitro*, *IDH*-2 mutated cell lines demonstrate increased glucose uptake,^10^ which suggests that this mutation could have led to our patient's worsening hypoglycemia. 2-HG effect on epigenetic modifications and metabolic adaptation suggests that pharmacologic inhibition of mutant *IDH* malignancies could result, at least in part, in oncogenic reversibility, and facilitate cellular stress induced by conventional cytotoxic agents.

At the molecular level, c-MYC induces overexpression of glucose transporters (GLUT), glycolytic enzymes and lactate dehydrogenase.^11^ Similarly, mutations in *p53* allows for unregulated expression of GLUT1 and GLUT4 transporters, increased activity of the glycolytic enzymes phosphofructokinase and phosphoglycerate mutase, and inhibit synthesis of cytochrome c oxidase 2. Furthermore, mutations in p53 allow for activation of HIF through PI3/AKT pathway to contribute to the Warburg effect.^3^ Reprogramming of glucose metabolism through *p53*, *IDH2* mutations, and MYC overexpression may have contributed to the fatal outcome in our patient characterized by refractory hypoglycemia, progressive lactic acidosis and rapid tumor growth. Furthermore, his disease may have progressed after an additional hit through mutant *IDH2* epigenetic modification.

There are limitations for our case. First, it is possible that, previous chemotherapy exposure in our patient, has resulted in genomic instability leading to myeloid rather than lymphoid restricted mutations. The possibility for potential myeloid derived mutations obtained from unfractionated whole marrow could have confounded putative role for *IDH* and *TP53* mutations in metabolic lymphoid resetting. Second, lack of functional, *in vitro,* assays to demonstrate mechanistic link between observed tumor mutations and metabolic cellular restructure limits our ability to render a direct pathogenic role for mutations in Warburg effect. However, our case highlights the importance of mutational analysis assisting in identification of phenotypic expression of cancer complications. Future directions include the investigation of targeting driver pathways that contribute to the Warburg effect. With ongoing development of IDH inhibitors, and possible access to rapid 2-HG serum testing, it may be feasible to explore the impact of these drugs on cancer patients exhibiting severe hypoglycemia, lactic acidosis and rapid tumor growth associated with metabolic resetting.

## Figures and Tables

**Figure 1:**
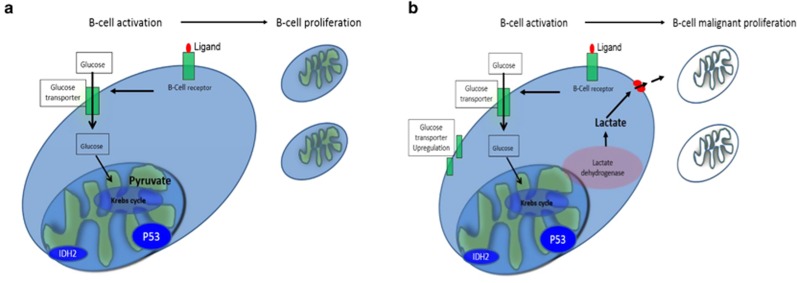
Glucose and energy metabolism in normal and transformed B cells. (**a**) Glucose intake is mediated by glucose transporters after B-cell receptor stimulation. Cells metabolize glucose to pyruvate to optimize energy expenditure. (**b**) Metabolome restructure is initiated by increasing glucose transporters (GLUT) and lactate dehydrogenase expression. Acquisition of Isocitrate dehydrogenase (IDH) and P53 mutations favor glycolysis and GLUT expression, respectively. High lactic acid production and hypoglycemia characterize Warburg resetting.

**Figure 2:**
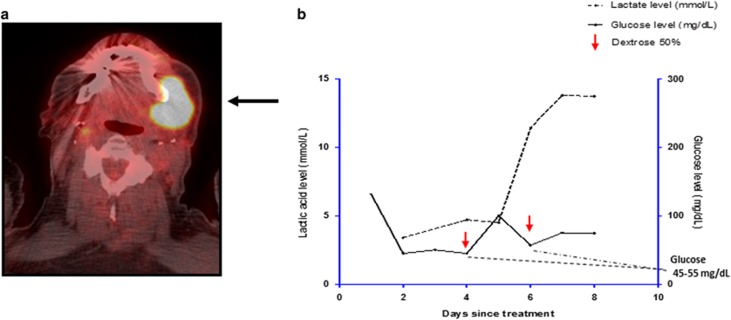
Submental PET-avid mass consistent with DLBCL and laboratory evidence for lactic acidosis and hypoglycemia. (**a**) Arrow shows large submandibular mass with intense uptake consistent with large cell transformation. (**b**) Refractory hypoglycemia without evidence for endocrine and infectious etiology. Marked lactic acidosis was observed during evolving hypoglycemia refractory to administration of intravenous high glucose concentration.
